# Gene Delivery into the Inner Ear and Its Clinical Implications for Hearing and Balance

**DOI:** 10.3390/molecules23102507

**Published:** 2018-09-30

**Authors:** Sho Kanzaki

**Affiliations:** Department of Otolaryngology Head and Neck Surgery, School of Medicine, KEIO University, Tokyo 160-8582, Japan; skan@keio.jp; Tel.: +81-35363-3827

**Keywords:** inner ear, hearing loss, gene delivery

## Abstract

The inner ear contains many types of cell, including sensory hair cells and neurons. If these cells are damaged, they do not regenerate. Inner ear disorders have various etiologies. Some are related to aging or are idiopathic, as in sudden deafness. Others occur due to acoustic trauma, exposure to ototoxic drugs, viral infections, immune responses, or endolymphatic hydrops (Meniere’s disease). For these disorders, inner ear regeneration therapy is expected to be a feasible alternative to cochlear implants for hearing recovery. Recently, the mechanisms underlying inner ear regeneration have been gradually clarified. Inner ear cell progenitors or stem cells have been identified. Factors necessary for regeneration have also been elucidated from the mechanism of hair cell generation. Inducing differentiation of endogenous stem cells or inner ear stem cell transplantation is expected. In this paper, we discuss recent approaches to hair cell proliferation and differentiation for inner ear regeneration. We discuss the future road map for clinical application. The therapies mentioned above require topical administration of transgenes or drug onto progenitors of sensory cells. Developing efficient and safe modes of administration is clinically important. In this regard, we also discuss our development of an inner ear endoscope to facilitate topical administration.

## 1. Introduction

Today, around 466 million people worldwide have disabling hearing loss, and 34 million of these are children [1]. It is estimated that over 900 million people will have disabling hearing loss by 2050 [1]. In children, hearing loss affects cognitive, language, and psychosocial development [1] whereas in the elderly hearing loss has been associated with cognitive decline [2] and depression [1]. While there are various causes of hearing loss, the most common cause is damage to the inner ear via trauma, exposure to ototoxic drugs, genetic mutations, viral infections, inflammation, or endolymphatic hydrops (Meniere’s disease). Sensorineural hearing loss comprises about 85% of all cases of hearing loss [1].

The inner ear comprises three main structures: the cochlea (hearing organ), the vestibule, and the semicircular canals (balance organ). Damage to the inner ear can have profound, long-lasting consequences, affecting not only hearing but also balance. As a result, researchers have focused on ways to mitigate or even reverse hearing loss. While cochlear implants are the intervention of choice, in recent years, the possibility of inner ear cell regeneration has received much attention as an alternative therapy for hearing loss. There are two main approaches to inducing regeneration of damaged cochlear hair cells: the injection of stem cells to replace dead cells and therapies that induce damaged sensory cells to regenerate. This review focuses on the latter, specifically the approach of using gene therapy to induce proliferation of inner ear cells. As this type of therapy requires topical administration of stem cells or progenitor cells, developing efficient and safe modes of administration is clinically important. In this regard, we also discuss our development of an inner ear endoscope that facilitates the topical administration of various therapeutic agents into the inner ear. Finally, we discuss the future road map for the clinical application of gene therapy in the treatment of hearing loss resulting from inner ear damage.

## 2. Results

### 2.1. Anatomy of the Inner Ear and Gene Delivery

**Figure 1:**
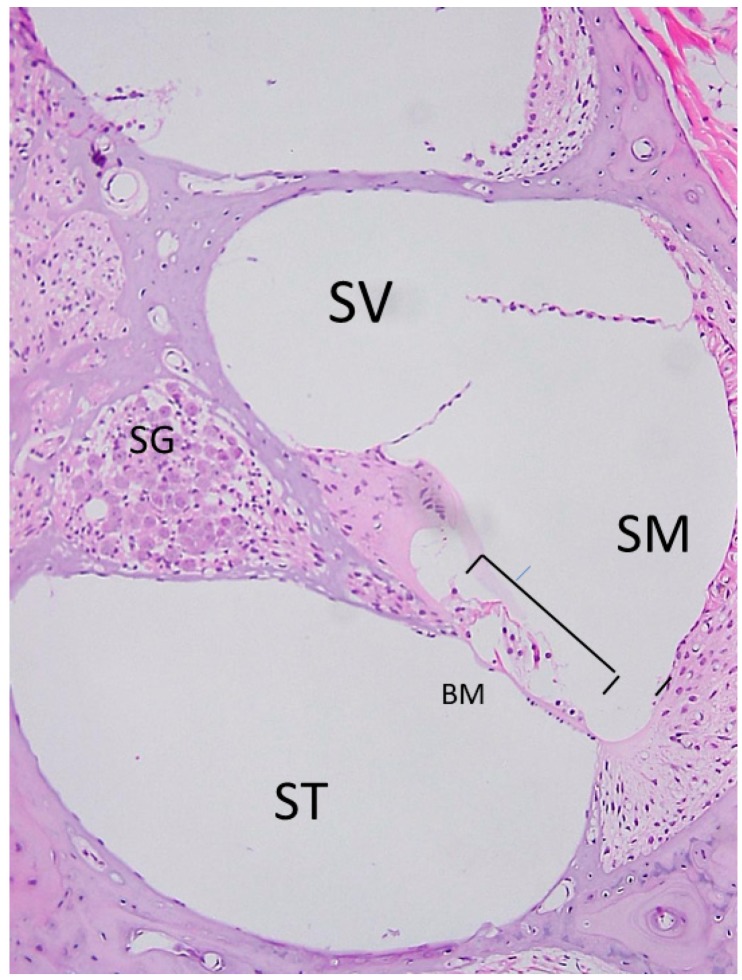
Photomicrographs of stained sections of the cochlea (hearing organ of the inner ear). Bracket delineates the organ of Corti, where hair cells and supporting cells (sensory epithelial cells) are located. The cochlea contains the scala media (SM), the scala tympani (ST), and the scala vestibuli (SV). SG, spiral ganglion; BM, basilar membrane.

The cochlea is the part of the inner ear that carries out the first steps of hearing. It comprises bony structures filled with two types of lymph fluid: perilymph and endolymph. The lymph transduces sound wave gathered by the outer ear and, via sensory hair cells, converts them to electrical signals that are transmitted first to spiral ganglion neurons (the first neuron of auditory nerve system) and then to the cochlear nerve and the brain. Damage to cochlear sensory hair cells results in sensorineural hearing loss, the most common type of hearing loss. One treatment strategy to mitigate this type of hearing loss is to inject Atoh 1 genes into the cochlea to induce hair cell regeneration [3]. As this part of the inner ear is essentially a liquid space, if a gene is administered to the cochlea, the gene can easily diffuse throughout the inner ear. Therefore, this must be done carefully, as large vibrations could damage the inner ear further when a hole in the inner ear is made for the device delivering the gene. The volume of both endolymph and perilymph and mode of administration are important (Figure 1).

### 2.2. Advantages and Disadvantages of Administering Vectors into the Inner Ear

In animal experiments, different types of vectors have been injected into the inner ear. Examples of viral vectors include adenovirus, herpes virus, Sendai virus, and adeno-associated virus vectors (Table 1). An example of a non-viral vector is liposomes. In clinics, adenovirus, herpes, and other viral infections of the inner ear have been observed [4]. Thus, there is a high risk of infection following injections of the viral vectors mentioned above. When administering the vector into the inner ear, a hole is made in the round window membrane or a hole is drilled in the otic capsule bone and then the vector is injected. As the inner ear is a sensory organ that senses vibrations, vibrations that occur while drilling holes or injecting drugs may result in mechanical damage to the inner ear. Thus, administration itself is a high-risk procedure that can lead to damage of auditory functions.

The inner ear is made up of three structures: the scala media, the scala vestibuli, and the scala tympani. Sensory hair cells and spiral ligaments that regulate ions are located on the scala media. Opening the otic capsule bone (lateral cochlear bone) to gain access into the scala media can potentially result in more damage than approaching through the round window membrane to gain access to the scala tympani or vestibuli, and in turn, the spiral ganglion. In the latter, hearing function is relatively preserved because hair cells are not damaged [5].

**Figure 2:**
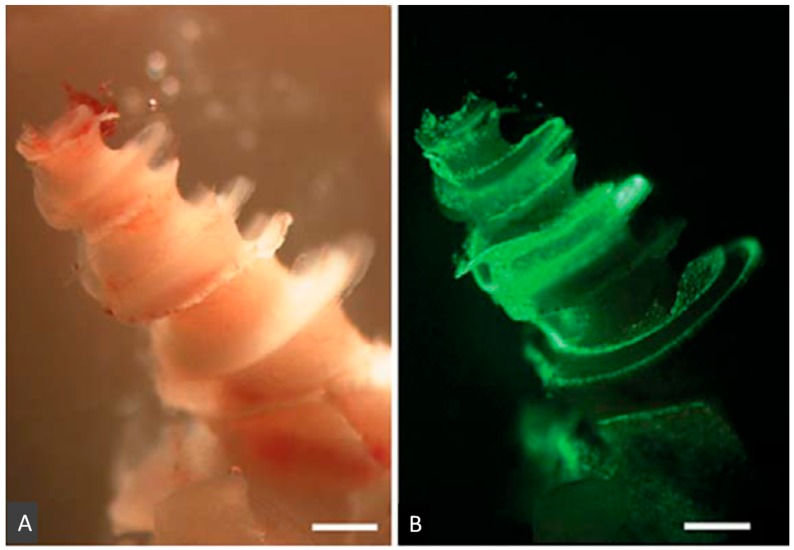
Photomicrographs of whole-mounted guinea pig cochlea showing the distribution of GFP-fusion protein after injection of GFP-fused Sendai virus vector (GFP-SeV/ΔF) into the scala media. Light (**A**) and fluorescent (**B**) images of the cochlea of a SeV-inoculated ear. Scale bars: 1000 μm (This figure was cited by reference [10] and permitted by S. Karger AG, Medical and Scientific Publishers.).

**Figure 3:**
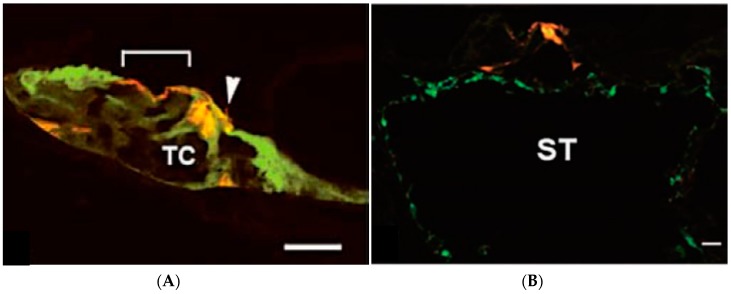
Photomicrographs of histological sections of guinea pig scala media after injection of GFP-SeV/ΔF (**A**) and after injection via a scala tympani approach (**B**). (**A**) Hair cells and supporting cells from the organ of Corti in an inoculated ear. An inner hair cell is indicated by an arrowhead, and an outer hair cell region is delimited by a bracket. Red, F-actin-stained with rhodamine phalloidin; green, GFP-SeV/ΔF-transfected cells. Scale bars: 10 μm. (**B**) Sensory epithelial cells and fibrocytes of the scala tympani. Numerous fibrocytes in the scala tympani are fluorescently labeled. BM, basilar membrane; SL, spiral limb; ST, scala tympani; TC, tunnel of Corti. Scale bars: 100 μm. (This figure was cited by reference [10] and permitted by S. Karger AG, Medical and Scientific Publishers.)

Different viral vectors, such as adenovirus vectors (ADV), can be used to transfect supporting cells (which are progenitors of hair cells), but not hair cells in mature cochlea through the scala media [6]. By using different viruses and different approaches, otolarygologists can deliver targeted therapies to different types of inner ear cells. While ADV can specifically be used to transfect supporting cells, Sendai virus can be used to transfect both hair cells and supporting cells via the scala media, and fibrocytes and spiral ganglion via the scala tympani (Figure 2 and Figure 3). There are variety of cells after inoculation of virus vectors (Table 1). The option of the viral vector depends on which cells are targeted. ADV [7], herpes virus vector (HSV) [8], Lenti virus vector (LV) [9], and Sendai virus vector (SEV) [10] need traumatic injection and transfect with mesenchymal cells in scala tympani or media through the opening of RWM (Table 1). In addition, because of their small size, adeno-associated virus vectors are able to pass through the round window membrane to reach the inner ear [11]. With adeno-associated virus vectors, since the round window membrane does not need to be opened to gain access to the inner ear, damage to the inner ear is avoided [12]. One drawback with adeno-associated virus vector (AAV)s, however, is that its s mall size limits the size of the transgene to be transfected. Its genome cannot exceed 5.2 kb in length [13]. ADV with inserts of 4.88 kb in length have been successfully constructed [14].

### 2.3. Regeneration of the Sensory Hair Cells

Unlike in birds, in mammals the reacquisition of hearing is difficult because in mammals most cells of the inner ear, including hair cells, do not regenerate [18]. For years, the first-line treatments for hearing loss were hearing aids and cochlear implants, as no therapeutic agents were available to facilitate the regeneration of damaged hair cells in patients with an irreversible inner ear disorder. Hearing aids and cochlear implants bypass the normal transmission route to the cochlea and targets instead the spiral nerve. Interestingly, vestibular hair cells, which act as “balancers,” possess some regenerative capacity, even in mammals [19]. What is it about vestibular hair cells that enable them to regenerate following damage? Although auditory hair cells possess little to no regenerative ability in mammals, would it possible to induce them to replicate? To answer these questions, further research on the regenerative capacity of cells responsible for balance not only will be important for developing treatments for dizziness and vertigo, but also may shed light on how to induce auditory hair cells to regenerate.

For decades, it has been known that auditory sensory hair cells of birds regenerate after trauma [18]. In birds, non-sensory supporting cells play an essential role in inducing regeneration of damaged sensory hair cells. Could the corresponding systems in mammals be leveraged to induce hair cell regeneration in mammals, including humans? In 2001, Ito et al. observed that neural stem cells grafted into the cochlea of newborn rats differentiated into cells resembling hair cells, even migrating to areas occupied by hair cells. In 2005, Izumikawa and co-workers discovered that introducing Atoh1, a gene encoding a key regulator of hair cell development, into non-sensory supporting cells in the cochlea induces the regeneration of hair cells in deaf guinea pigs [3]. However, why is hearing improvement not observed in all animals? Perhaps the answer to this question depends on the type of cells capable of regenerating. In the studies described above, the regenerated hair cells were not inner or outer hair cells.

**Figure 4:**
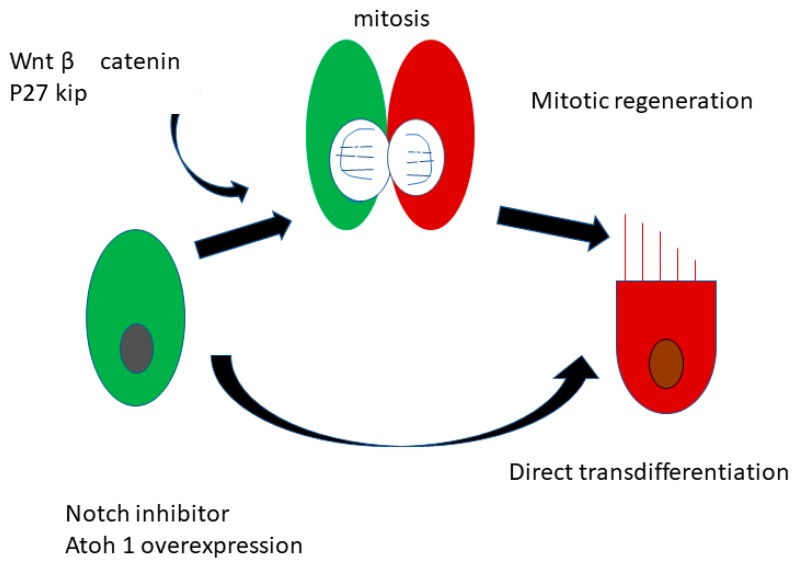
Schematic of the mechanisms underlying inner ear hair cell regeneration. There are two theories concerning hair cell regeneration: (1) direct of transdifferentiation from supporting cells (SCs) to new hair cells (HCs) (green), and (2) proliferation of SCs and mitotic regeneration (red).

These research studies serve as the foundation of a new branch of hearing research—that of inner ear regeneration medicine. Since then, much work has been done to identify the specific mechanisms responsible for the differentiation of progenitor cells to auditory or vestibular hair cells, and now responsible signaling pathways and regulatory sequences are known (Figure 4). 

Great advances in basic and clinical hearing research have been seen in the 21st century. Hair cells, spiral neurons, spiral ligaments, and stria vascularis, among others, are candidate targets of regenerative medicine aimed at treating sensorineural hearing loss. In the following section, I summarize current strategies for regenerative therapies: regeneration of cells remaining in the inner ear using genes or drugs (gene therapy, drug treatment); and replacement of damaged inner ear cells (cell therapy). 

As mentioned, birds, as well as fish and amphibians, possess the capacity to regenerate injured hair cells via direct and indirect trans-differentiation of progenitor cells [18]. Therefore, in mammals, studies on the regeneration of hair cells have been based on research done on the mechanisms underlying regeneration of inner ear hair cells in birds, fish, and amphibians. The basic strategy for promoting hair cell proliferation and differentiation in deaf mammals, therefore, is to induce regeneration of hair cells by first initiating the transdifferentiation of supporting cells to hair cells.

In order to accomplish this, it is necessary to know the key signal pathways responsible for mediating the development (differentiation and proliferation) of these cells. In postnatal hair cells, the cell cycle stops and remains in the resting phase. In 1999, Lowenheim and colleagues identified a substance, p27 kip, responsible for preventing these cells from dividing and proliferating [20]. They also discovered that cochlear supporting cells specifically express p27 kip, which is a cyclin-dependent kinase inhibitor. They hypothesized that inhibition of p27 kip might cause supporting cells to re-enter the cell cycle, and thus regain the ability to proliferate. Knocking out p27 kip in mice did indeed enable these cells to do just that. This finding paved the way for using similar pathways for inducing hair-cell regeneration in mammals [21]. One drawback to knocking out p27 kip is that it increased the risk of those mice of developing cancer.

Other signaling molecules involved in regulating the cell cycle have also been analyzed. For example, p19 Ink4d [22] and retinoblastoma (Rb) [23] have also been found to be involved in cell cycle regulation. The Wnt/catenin signal, which can also regulate the extent of proliferative hair cell regeneration, is also involved [24] (Figure 4).

It is conceivable that inner ear progenitors (stem cells or supporting cells) might differentiate into hair cells. The Atonal homolog 1 (*Atoh 1*) gene is necessary for the generation of hair cells of the inner ear [25]. In ex vivo [26] and in vivo [3] organ cultures of mouse inner ear, genes, such as *Atoh1*, can be introduced into candidate progenitor cells using adenoviral vectors. Using adenoviral vectors, Izumikawa et al. introduced *Atoh1* into inner ear supporting cells of a guinea pig that was made experimentally deaf by drug application into one ear. The untreated ear did not have any new or regenerated hair cells. The treated ear, however, had new “hair cells” that possessed the ability to regenerate [3]. Under electron microscopic examination, it was apparent that *Atoh1*-transfected supporting cells had transdifferentiated into hair-cell-like cells [3]. 

More efficient and safer viral vectors are now available for transfecting genes into the inner ear. For example, Sendai virus vectors and adeno-associated viral vectors have equal or higher gene transfer distribution to the inner ear than adenoviral vectors and are safer [10,12]. 

### 2.4. Preventing Degeneration of Inner Ear Spiral Neurons and Neuron Regeneration

Spiral ganglion neurons are primary neurons of the auditory system, and when hair cells are damaged, spiral ganglion neurons are also at risk of being damaged. Spiral ganglion neurons that degenerate following damage to inner ear hair cells are called secondary spiral ganglion neurons or residual spiral ganglion neurons [27]. As these are the same cells targeted by gene therapy for treatment of hearing loss, hearing recovery depends on the presence of these cells. These cells are also thought to play an important role in the success of cochlear implants. Although still controversial, the notion that the number of remaining spiral ganglion neurons correlates with the treatment outcome (i.e., word intelligibility score) of cochlear implants has been recently reported [28]. 

Following inner ear damage, it is important to preserve as many surviving spiral ganglion neurons as possible. Some studies have demonstrated that neurotrophic growth factors can stave off the degeneration of spiral ganglion neurons. One experiment compared the ability of electrical stimulation and glial-derived neurotrophic factor (GDNF), alone and in combination, to enhance the survival of residual spiral ganglion neurons in a guinea pig model of deafness [27]. Combination electrical stimulation/GDNF gene therapy significantly prevented spiral ganglion neuron degeneration compared to electrical stimulation or GDNF therapy treatment given alone [27]. In a similar experiment involving deaf guinea pigs, administration of brain-derived neurotrophic factor (BDNF) gene into the cochlea improved electrically induced electrical auditory brainstem responses (eABR), and specifically, neurological function [29].

### 2.5. Development of Endoscope for Topical Administration of Transgenes into Inner Ear

Applying therapeutic agents directly into the inner ear is the desired and most effective method to treat inner ear disorders. However, doing so can be problematic, as the structures are small and delicate and hence easily damaged. The novel endoscope we developed is capable of detecting round window obstructions due to the presence of a pseudo-membrane over the round window (30% of clinical cases have this problem; [30]). This is a critical issue for injections of gene and drug therapies. If a pseudo-membrane is found, we can open the membrane, and then inject transgene or viral vectors. In addition, the endoscope can be used to observe the inner ear through a small hole in the tympanic membrane. This route is easily accessible, and administering therapeutic drugs through this route is less invasive. 

To facilitate inner ear injections, to guide placement of injections, and to enable clinical observation of inner ear structures during the injection procedure, we developed a specialized endoscope that allows drug or gene administration to the inner ear [31]. This device enables clinicians to observe the round window during the procedure [31]. In order to observe the inner ear through the middle ear via a slit in the eardrum, it is necessary to further reduce the diameter of the endoscope so as not to invade the tympanic membrane. Our novel otoendoscopy device, which comprises a scope, light guide, and catheter channel for injecting genes or drugs, permits discrete and controlled inner ear injections, which is necessary for delivering gene therapy to inner ear structures [31].

### 2.6. Problems Associated with Inner Ear Gene Delivery for Clinical Use

So far, we have described fundamental research results concerning inner ear gene delivery. Another issue to consider is the problem of predicting the pathologies of deafness in individual patients. For example, therapy depends on two things: (i) whether only hair cells are lost and supporting cells remain, and (ii) whether both hair cells and supporting cells are lost. In the first scenario, treatment with a hair cell differentiation-inducing agent is indicated. However, in the second scenario, cell therapy is indicated. Thus, if physicians can determine the status of inner ear cells (i.e., which cells remain), they can choose the most effective treatment with the best use of patient’s cells. At this time, the lack of a method that can determine the status of inner ear cells hinders progress in cell regeneration and replacement therapies for clinical application. For cases in which much time has passed since the onset of severe hearing loss, as for example, in genetic hearing loss, it is likely that the sensory epithelial cells of the organ of Corti are probably completely wiped out. This kind of case is a good candidate for cell therapy. We need to accurately determine the pathological status of inner ear cells before treatment. While our endoscope can achieve this to some extent, in clinics, less invasive and high-resolution imaging such as magnetic resonance imaging (MRI) [32] or optical coherence tomography (OCT) [33] can bridge the gap by providing more information about inner ear pathology. 

To date, there are a limited number of published studies on the use of gene therapy for genetic hearing loss. This is ironic, as gene therapy is indicated for this type of hearing loss. 

In cases with advanced hearing loss where the patient cannot hear, even with hearing aids, cochlear implants are indicated. The most feasible clinical application of gene therapy for hearing improvement would be combination treatment comprising administration of neurotrophic factor transgenes in conjunction with cochlear implants. 

After repair and regeneration of inner ear cells, the auditory network should be reconstructed and hearing improvement should be observable. However, simply hearing sounds may be clinically insufficient. Since understanding languages is important, hearing rehabilitation is essential in order to compensate for the lack of hearing. Hearing rehabilitation is typically implemented after wearing hearing aids and after implantation of cochlear implants. Electrical stimulation delivered in conjunction with neurotrophic factors supports neuronal elongation and promotes the hearing network system and rehabilitation [27].

### 2.7. Genetic Hearing Loss

More than 100 types of genetic hearing loss exist [34]. Patients with genetic hearing loss are potential recipients of gene therapy interventions. However, in most congenital hearing loss cases, morphological changes in the inner ear limit the time window for successful transduction, and in turn, functional recovery [35]. The results of one study support this notion. In that study, sh2/sh2 zygotes of deaf shaker mice were transfected with a bacterial artificial chromosome transgene containing the Myo15a gene [36], which encodes unconventional myosin, and cochlear cell morphology and hearing function were assessed 2, 4, and 6 months later. Myo15a transfection into zygotes was capable not only of maintaining normal hair cell morphology but also was capable of conferring stable hearing function to these Myo15a mutant mice for as long as 6 months [35].

Recently, perinatal injection of the Gjb2 gene into the cochlea using adeno-associated virus 5 (AAV5) was shown to preserve some cochlear structures and improve hearing in a conditional Gjb2 knockout mouse model [37]. Adeno-associated virus 2 (AAV2) harboring a Ush1c transgene also restores auditory and vestibular function in a mouse model of Usher syndrome [38] These studies demonstrate the potential of transgene therapy for restoring hearing in cases of genetic hearing loss. 

## 3. Conclusions

Great strides have been made in the field of inner ear regenerative medicine in terms of gene delivery therapies for the treatment of sensorineural hearing loss and the prevention of hearing degeneration in patients with genetic hearing loss. Basic research on the induction of differentiation of cochlear progenitor cells and prevention of auditory neuronal degeneration support the use of these methods for clinical applications. To make full use of these therapies, accurately determining the pathological status of inner ear cells is required. Researchers need to develop a practical method for clinicians to determine this status. One step in this direction is the use of a specialized endoscope, like the one developed in our clinic, that is miniaturized and allows clinicians to directly observe the environment inside the inner ear.

## Figures and Tables

**Table 1:** Comparison among viral vectors in inner ear.

Viral Vector	Animal	Transfected Cells		RW Penetration	References
		via SM	via RW		
ADV	guinea pig	SC, SV, SL	mesenchymal cells in SV/ST	NO	Raphael, Y. 1996 [7]; Weiss, M.A. 1997 [15]; Ishimoto, S. 2002 [5]
AAV	mouse	IHC, OHC, SC, SG, SL, SV	IHC, OHC, SC, SG, SL, SV	YES	Iizuka, T. 2008 [12]; Shu, Y. et al., 2016 [16]
AAV	guinea pig	Not reported	spiral limbus, SL, SG, organ of Corti		Lalwani, K. 2000 [17]
HSV	guinea pig	Not reported	fibrocytes (types I, II, IV), mesenchymal cells, HCs	NO	Derby, M.L. 1999 [8]
LV	guinea pig	Not reported	mesenchymal cells in ST		Han, J.J. 1999 [9]
SEV	guinea pig	IHC, OHC, SC,	mesenchymal cells in ST	NO	Kanzaki, S. 2007 [10]

Different approaches indicate different distributions of transfections in same virus vector. ADV; Adenoviral vector, AAV; Adeno-associated virus vector. HSV; Herpes virus vector, LV lenti virus vector, SEV; Sendai virus vector, IHC inner hair cell, OHC outer hair cell, SC; supporting cell, SG; Spiral ganglion, SL; Spiral ligament, SM; Scala media, ST; Scala tympani, SV; Scala vestibule, RW; round window.
